# Differences in the Early *In Vitro* Development of Preimplantation Human IVF Embryos Which Go on to Develop Congenital Heart Disease

**DOI:** 10.3390/jcdd12090370

**Published:** 2025-09-19

**Authors:** Sophie Markham, Alison Campbell, Sue Montgomery, Iain M. Dykes

**Affiliations:** 1 Department of Pharmacy and Biomolecular Sciences, Liverpool John Moores University, Byrom St., Liverpool L3 3AF, UK; 2 Care Fertility Manchester, 108-112 Daisy Bank Road, Victoria Park, Manchester M14 5QH, UK; alison.campbell@carefertility.com (A.C.); sue.montgomery@carefertility.com (S.M.); 3 Department of Biosciences, University of Kent, Canterbury CT2 7NZ, UK; 4 Liverpool Centre for Cardiovascular Science, Institute for Health Research, Liverpool John Moores University, Liverpool L3 3AF, UK

**Keywords:** morphokinetics, fertility treatment, diagnostics, congenital heart disease, retrospective analysis, embryology

## Abstract

There is a clinical need for improved antenatal diagnosis of congenital heart disease (CHD). Increasing numbers of children are born to parents undergoing fertility treatment. We asked whether time-lapse imaging of *in vitro* preimplantation development provides diagnostic information. We performed a retrospective multicentre analysis of morphokinetic data from patients undergoing fertility treatment. A total of 96/18,799 CHD cases were identified (rate: 0.51%). Thirty-two were included in the analysis and stratified into three cohorts: complex CHD (n = 7), mild CHD (n = 11) and murmur only (n = 14). Comparison to a large unmatched control group (n = 352) revealed no differences in time of preimplantation developmental events but suggested an increase in cleavage synchronicity during the third cell cycle of mild CHD embryos. Pairwise comparison to matched controls revealed a delay in mild CHD embryos relative to controls in reaching the morphokinetic timepoints fading of pronuclei, 2-cell stage and 4-cell stage together with a possible increase in duration of blastulation in complex CHD. Our data raises the possibility that screening of preimplantation embryos at fertility clinics could reduce the rate of CHD. However, these results are preliminary, and further work is required to confirm the findings in a larger study.

## 1. Introduction

Congenital heart disease (CHD) is the most prevalent birth defect, with estimates of disease incidence varying between 0.68% and 1.1% of live births in the general population [1,2,3,4]. Despite advancements in cardiovascular medicine and surgery enabling most patients to reach adulthood, CHD is still the most frequent cause of infant death from birth defects and continues to impose a heavy disease burden as well as financial and emotional stress on families worldwide [5]. CHD is a heterogeneous disease which encompasses many subtypes. Cyanotic disease is the most serious form of the disease, involving multiple, often complex lesions which may threaten the life of the newborn child and generally require surgery or other interventions [6]. Cyanotic disease accounts for approximately 1/5 to 1/4 of CHD cases in the general population [1,3], with the remainder being the milder acyanotic form, generally involving a single lesion [7]. Some acyanotic forms may heal naturally without treatment, while others may require cardiology procedures such as angioplasty [7].

A number of studies have suggested that fertility treatment is linked to increased risk of developing birth defects. ICSI treatment has been reported to double the incidence rate of major birth defects [8]. Meta-analyses have repeatedly concluded that embryos conceived with both IVF and ICSI methods are at an increased risk of developing CHD, with reported incidence rates ranging from 1.3% to 3% [4,9]. However, such studies are complicated by the heterogeneity of CHD, and while some studies have suggested that this risk is specific to acyanotic forms of CHD such as ventricular septal defect [4,9,10,11], others report increased risk of cyanotic disease such as outflow tract defects [12]. Echocardiography indicates that the hearts of six month old foetuses derived from fertility treatment exhibit specific changes associated with cardiac remodelling [13].

Prenatal diagnosis improves clinical outcomes for CHD. A universal screening programme is offered to all pregnant women in the UK, and CHD is normally diagnosed during the foetal anomaly ultrasound screen performed at approximately 20 weeks. However, about 50% of CHD cases remain undiagnosed until birth [14,15]. Many *in vitro* fertilisation (IVF) clinics offer preimplantation genetic tests for aneuploidy (PGT-A). Down’s syndrome and other aneuploidies are commonly associated with CHD and account for 13% of all CHD cases, while copy number variants, including 22q11 deletion, account for 15% [16]. PGT-A is not routinely offered because such technologies require trophectoderm biopsy and genetic testing, which are not available in all fertility clinics, and there are concerns regarding the accuracy of the test due to mosaicism [17]. Preimplantation genetic testing for specific causative genes associated with heritable adult cardiovascular diseases has been reported [18]. However, the applicability of this to CHD may be limited because only a small minority (12%) of CHD cases result from single gene mutations [16].

Time-lapse imaging of the preimplantation embryo is used in many IVF clinics for embryo selection through a detailed analysis of developmental events known as morphokinetics [19,20,21]. Both chromosomal and segmental aneuploidies result in specific developmental delays which may be detected by morphokinetic analysis [21,22,23]. When such data are maintained until after birth of the child, this presents an opportunity to assess the correlation between variation in early developmental events and the subsequent risk of developing congenital disease. As well as providing fundamental insights into developmental processes, we postulated whether such data could be used to improve prenatal screening for CHD.

While a minority of CHD phenotypes have an identified genetic cause and a clear molecular/cellular mechanism, the cause of the majority (60% of cases) remains unknown [16]. No events prior to gastrulation are known to influence the development of the cardiovascular system nor any other organ. However, recent evidence suggests that the blastomeres of the early embryo are not equivalent and make an unequal contribution to the epiblast (embryo proper) [24]. This suggests that the earliest events in embryonic development could contribute to later patterning events important in determining morphogenesis of organs such as the heart. In support of this hypothesis, a retrospective analysis of morphokinetics data identified putative delayed blastulation in embryos giving rise to newborns with a range of isolated congenital anomalies [25], although none were cardiovascular. The same study also found delayed blastulation in embryos giving rise to gestational diabetes, while those born pre-term were associated with longer cleavage-stage cell division cycles [25].

In this study, we performed a retrospective analysis of morphokinetics data from embryos known to have produced children affected by CHD. We performed two analyses: one against a larger unmatched cohort of embryos and the second against a smaller matched cohort. This latter analysis was required because relatively minor clinical factors, including choice of culture medium and even how often the incubator door is opened, have been demonstrated to impact upon human preimplantation embryo morphokinetic timings [26,27,28].

Our analysis must be treated with caution due to the low power of the analysis but raises the possibility that specific preimplantation events may be linked to the risk of developing CHD.

## 2. Materials and Methods

### 2.1. Study Population

A retrospective multicentre analysis was performed on anonymised data obtained from Care Fertility clinics in the UK and Ireland. All patients underwent fertility treatment (*in vitro* fertilisation or intracytoplasmic sperm injection) which resulted in a live birth between January 2012 and April 2022.

### 2.2. Ethical Approval

Approval for the research was granted by Care Fertility’s multidisciplinary Research and Innovation Board with representatives from the governance, scientific and medical leadership teams. Specific ethical approval was not deemed necessary as the research was conducted using fully anonymised data collected during routine licenced fertility treatment, with robust data governance and no additional interventions or data collection. The investigation conforms to the principles outlined in the Declaration of Helsinki. All patients provided informed consent for non-contact research via a Human Fertilisation and Embryology Authority (HFEA) consent form.

### 2.3. Fertility Treatment and Embryo Culture

All data were collected as a part of routine fertility treatment at a Care Fertility clinic. Laboratory techniques are standardised across all Care Fertility clinics. Details regarding oocyte collection, intracytoplasmic sperm injection (ICSI), *in vitro* fertilisation (IVF) and embryo culture at Care Fertility clinics have been previously described [20,21].

### 2.4. Insemination and Time-Lapse Imaging and Morphokinetic Data Collection

Morphokinetics is a term to describe the timing of morphological events during embryogenesis. A detailed protocol for morphokinetic analysis at Care Fertility clinics has been previously published [20]. Immediately following ICSI or at fertilisation confirmation after IVF, embryos were transferred to the EmbryoScope time-lapse incubator (Vitrolife, Gothenburg, Sweden) and cultured for 5–6 days prior to intrauterine transfer to the mother. For ICSI, a single sperm was injected into each mature oocyte, and for IVF, oocytes were mixed with a fixed concentration of motile, washed sperm. The decision as to whether to perform ICSI or IVF is primarily driven by sperm numbers and quality or by previous fertility treatment outcomes. Time-lapse images were collected every 5–10 min for the duration of culture. These images were annotated by registered clinical embryologists employed at Care Fertility and trained in this technique to record the time of key developmental events. Care Fertility embryologists employ a centralised annotation protocol and rigorous quality control to ensure standardised annotation. Annotations are logged using the EmbryoViewer image analysis software in use at the time of treatment, up to V7 (Vitrolife, Gothenburg, Sweden). 

### 2.5. Morphokinetic Data Analysis

Time zero is manually set. For ICSI procedures, the midpoint of the ICSI procedure is used, while for IVF, time zero is recorded as the time the sperm is added to the dish containing the oocytes. The time of key morphological events occurring during the first 5–6 days of life were recorded, up until fresh embryo transfer or cryopreservation. Abbreviations used in this paper are standard within the field, and the reader is referred to previous descriptions [21]. These include fading of pronuclei (tPNf), early cleavage cell divisions (resulting in 2-cell to 8-cell embryos; t2-t8) and the post-cleavage stages of morula formation (tM), start of blastulation (tSB) and full blastocyst formation (tB). We did not analyse the milestones, start of compaction (tSC), expanded blastocyst (tEB) and hatched blastocyst (tHB), because the dataset was incomplete for these variables, indicating that they were not reached, observed or that the annotation was omitted. Double embryo transfer and twin births from single embryo transfer were also excluded from the analysis.

### 2.6. Identification of the Large Control Cohort

A sample of 352 patients was selected from 7 Care Fertility clinics in the UK and Ireland (Manchester, Dublin, London, Northampton, Nottingham, Sheffield and Tunbridge Wells) who underwent fertility treatment between January 2013 and November 2018. Inclusion criteria were (i) fresh embryo transfer (not frozen), (ii) CHD-unaffected live birth and (iii) complete morphokinetics dataset available (tPNf to tB).

### 2.7. Identification of the CHD and Matched Control Groups

Records of live births were obtained from 7 Care Fertility clinics in the UK and Ireland (Manchester, Northampton, Nottingham, Bath, Birmingham, Sheffield and Dublin). A total of 18,799 patients were included in the analysis. To identify CHD cases, the clinic live birth outcome database was examined. All live births resulting from assisted conception treatment must be reported to the Human Fertilisation and Embryology Authority (HFEA) in the UK, and the detail required includes the occurrence of any birth defects. The terms ‘heart defect’ or ‘heart murmur’ reported on this form were taken as evidence of CHD. Any further details regarding the medical history of the baby, parents or donors given on the form were recorded. From this group, cases for morphokinetic analysis were selected based on (i) availability of morphokinetic data, (ii) birth resulting from a single embryo transfer and (iii) live birth resulting in a single child. To identify matched controls for each case, the following parameters were applied in this order: (i) a single embryo transfer resulting in a live birth, (ii) treated at the same clinic, (iii) the same method of fertilisation and (iv) as close as possible to the date of embryo transfer, ideally the same month/year (all except one were within 2 months of the treatment date).

### 2.8. Data Stratification

CHD cases were allocated to one of three cohorts using the following criteria. The complex CHD cohort contained the most seriously affected patients. These patients generally have multiple cardiovascular phenotypes or have a single more serious phenotype. Some required surgery or died peri- or postnatally, indicating probable cyanotic CHD. The mild CHD cohort includes patients who generally have a single phenotype that is unlikely to be life-threatening (acyanotic) and most likely would not require surgery. Examples include isolated ventricular septal defect and patent foramen ovale. Included in this cohort are patients reported to have a heart defect but with no further details provided on the live birth outcome form. Finally, the murmur cohort contains patients reported to have a heart murmur but whose live birth outcome form does not report any other defect. This cohort are likely to be the least affected patients.

### 2.9. Statistical Analysis

Statistical analysis was performed using Biorender. Graphs and diagrams were also prepared using Biorender. Details of statistical analysis are provided in each figure legend.

## 3. Results

### 3.1. Study Design

**Figure 1:**
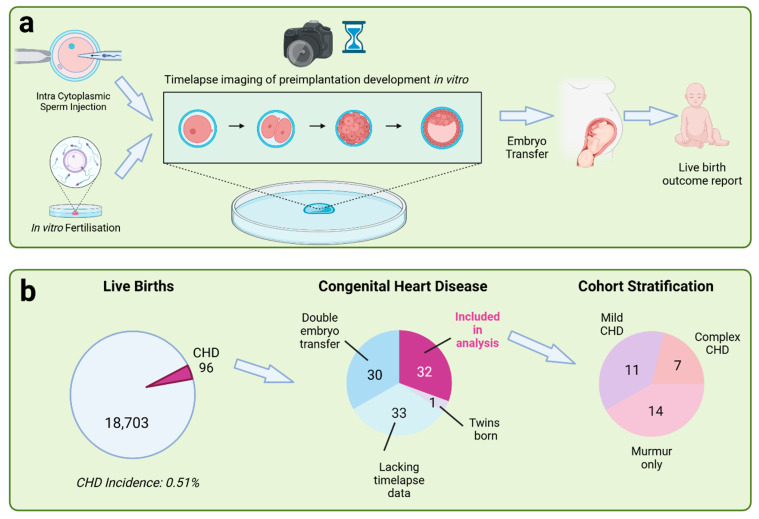
Overview of the study. (**a**) Embryos were transferred to the Embryoscope incubator immediately after intracytoplasmic sperm injection (ICSI) or following fertilisation confirmation (presence of pronuclei) the day after *in vitro* fertilisation (IVF). Time-lapse images were taken during the first 5–6 days of development. Embryos were then transferred into the uterus of the mother. Following birth, a live birth outcome report was submitted detailing the health of the child. (**b**) A retrospective analysis was performed. From a total of 18,799 live births across 7 clinics, 96 cases of congenital heart disease were identified. Sixty-four cases were rejected due to either lack of imaging data, births resulting from a double embryo transfer or birth of a twin. This left 32 cases in the study group. These cases were stratified into three cohorts based on the severity of the disease: murmur, mild CHD and complex CHD.

This is a retrospective multicentre analysis of data obtained from a large database containing records of 18,799 live births resulting from treatment at Care Fertility clinics in the UK and Ireland. Many embryos at Care Fertility clinics are cultured in an Embryoscope incubator for the first 5–6 days of life, prior to being transferred into the mother’s uterus (Figure 1a). The Embryoscope contains a built-in microscope and camera which takes a photograph approximately every 5–10 min. Trained clinical embryologists can then use these photographs to determine the time of key morphological events during development such as cell cleavages, morula and blastula stages; these are known as morphokinetic data. Following birth, the clinic submits an HFEA live birth outcome form containing details of the birth and health of the newborn. We were able to identify children affected by congenital heart disease using this report (Figure 1b) and then to go back and obtain their morphokinetics data. We compared these data both to a larger group of CHD-unaffected control embryos and to a smaller group of controls, in which each case was matched to a single control as closely as possible based on treatment variables.

### 3.2. Effect of Fertilisation Method on Morphokinetics of Preimplantation Development

We first used an unmatched control cohort of unaffected live births to investigate whether any variables related to fertility treatment affected the preimplantation development of embryos.

A cohort of 352 embryos were identified arising from treatments across multiple sister centres, all of which involved transfer of a single, fresh (rather than a frozen) embryo and which resulted in birth of a CHD-unaffected newborn (Table 1, line 1; Supplementary Table S1). Over three quarters of these births resulted from ICSI treatment (78.1%), with the remainder from IVF (21.9%). 14.2% of births were derived from a donor oocyte, while 8.5% used donor sperm.

**Figure 2:**
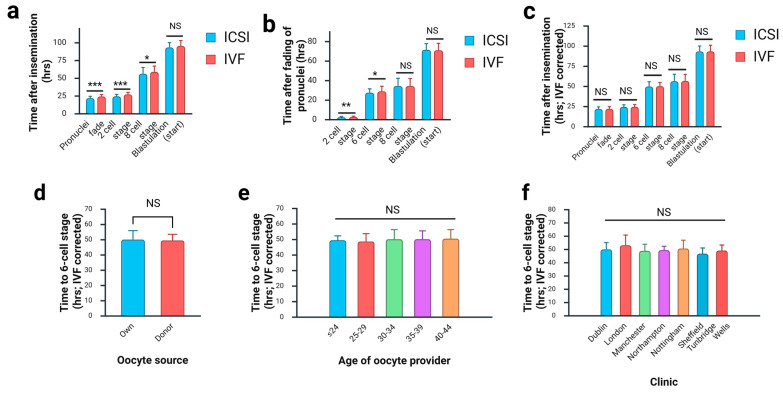
Effect of fertility treatment variables on embryo development. Analysis of unaffected control group. (**a**) IVF-derived embryos were significantly delayed relative to ICSI-derived embryos when measured from time of insemination. Selected developmental milestones shown. (**b**) When time of pronuclei fading is taken as the baseline, we continue to observe a significant delay in IVF-derived embryos at earlier stages. (**c**) Values for IVF-derived embryos have been corrected by subtracting the difference between median values for time post insemination for ICSI-derived and IVF-derived embryos. A conversion factor was calculated for each developmental milestone (Table 2). This removes the difference between ICSI-derived and IVF-derived embryos, indicated by the non-significant test result. (**d**) There is no difference in developmental timing between embryos derived from a donor oocyte and the mother’s own. (**e**) There is no difference in developmental timing between embryos derive from oocytes from providers of different ages. (**f**) No effect of treatment clinic was observed. Graphs show mean ± SD. Non-parametric statistical testing was used: (**a**–**d**) Mann–Whitney U-test; (**e**,**f**) Kruskal–Wallis test. *** = *p* < 0.0005; ** = *p* < 0.005; * = *p* < 0.05; NS: not significant. In each comparison, at least one group is not normally distributed (Shapiro–Wilk test).

We first looked at the effect of fertilisation method on the data. ICSI and IVF are different insemination methodologies, and for this reason, it is difficult to standardise time zero in morphokinetic data. Others have noted that IVF-derived embryos are delayed at early morphokinetic milestones relative to ICSI-derived embryos [20,29]. This is postulated to be because time zero is set when sperm are added to the dish in an IVF procedure, yet sperm require a certain amount of time to reach and penetrate the oocytes after this. In an ICSI procedure, sperm are injected directly into the oocytes, and time zero is set midway through this procedure. Our data confirm this (Figure 2a). We observe a highly significant delay in IVF-derived embryos at the earliest developmental mileposts such as time of pronuclei fading and time to reach the 2-cell stage. By the 8-cell stage, there is still a delay, but the difference is less significant, and by the start of blastulation, we no longer observe a significant difference (Figure 2a).

It is therefore clear that the data must be normalised to allow a direct comparison between IVF-derived and ICSI-derived embryos. Others have reported that the time of pronuclei fading can be taken as the baseline against which other times are compared [29]. However, when we plotted time elapsed since time of pronuclei fading, we continued to observe a significant difference between the two groups at early timepoints (Figure 2b).

We therefore calculated the difference in the median values for all embryos in the unaffected cohort between IVF-derived and ICSI-derived embryos for each developmental milestone (Table 2). We found that the delay was not constant but varies between developmental milestones. When this value was subtracted from IVF-derived embryos prior to plotting, we no longer observed a significant difference (Figure 2c). This transformation was applied in subsequent analyses to all data, including mixed IVF and ICSI embryos.

We next looked at additional fertility clinic variables. For these analyses, we examined a single developmental milestone approximately midway through the time period under investigation: time from insemination to the 6-cell stage. IVF-derived embryo data was corrected as above. We did not observe a significant effect of using a donor oocyte (Figure 2d), of age of oocyte provider (Figure 2e), nor of the clinic at which the procedure was performed (Figure 2f). Thus, it appears that the choice of IVF against ICSI is the only variable of those investigated affecting development of control unaffected embryos.

### 3.3. Identification of Congenital Heart Disease Cases

We identified 96 CHD cases from a study population of 18,799, giving a CHD incidence rate of 0.51%.

We rejected those embryos derived from a double embryo transfer (30), those that gave rise to twins (1) and those for which morphokinetics data was missing (33), leaving a total of 32 embryos included in subsequent analyses (Figure 1b). Table 3 lists the cardiovascular phenotype of each case within this cohort together with details of any other reported birth defects and of any genetic or chromosomal testing. These data are derived from the live birth outcome form, and the level of detail reported varies considerably between cases (Table 3).

CHD is a heterogeneous disease which arises from multiple causes, results in a wide range of specific phenotypes and can vary greatly in severity. About 25% of cases result in cyanotic disease which can be life threatening and often requires surgical intervention. At the other end of the spectrum, many heart murmurs are benign. For this reason, we decided to stratify the dataset into three cohorts reflecting decreasing levels of severity: complex CHD, mild CHD and murmur (Figure 1b; Table 1 and Table 3). Details of the criteria used to stratify are given in the methods.

### 3.4. CHD Embryo Development Against Unaffected Controls

In the first analysis, we compared the morphokinetics data of our three CHD cohorts (Supplementary Table S2) to that of the unmatched unaffected control group (Supplementary Table S1). All cohorts contained a mix of IVF- and ICSI-derived embryos; therefore, a correction was applied to data from IVF-derived embryos as described above. We use the terminology defined in Ciray *et al.* [30] here to describe events in preimplantation development.

**Figure 3:**
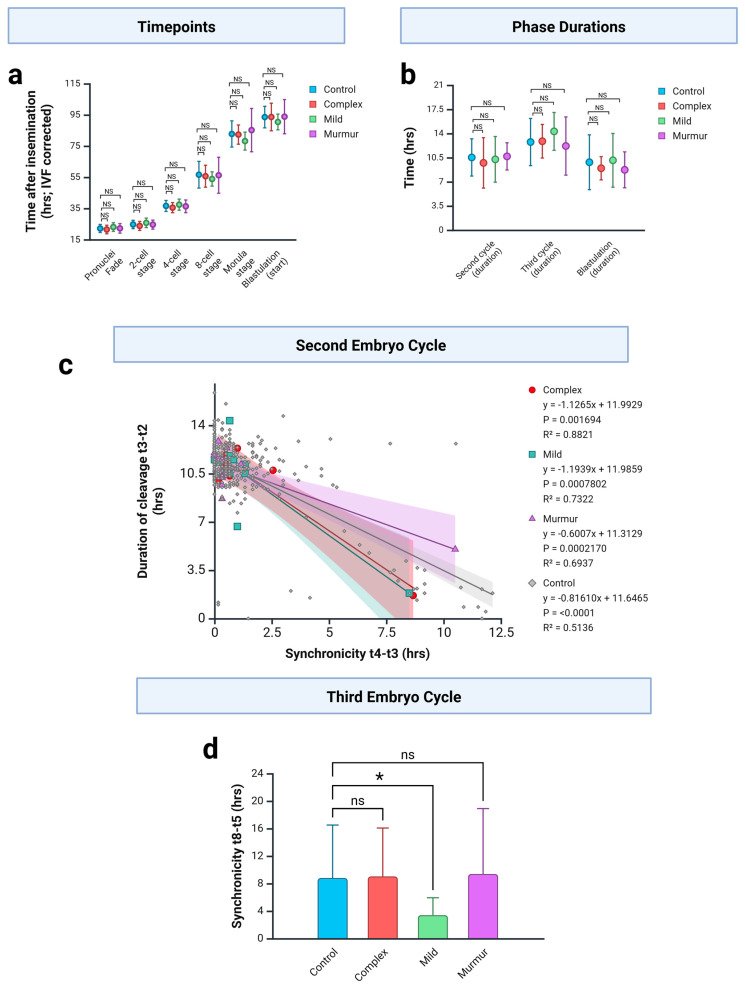
Comparison of CHD embryos to healthy controls suggests changes in cell cycling. (**a**) Time from insemination to key developmental milestones. IVF embryo values were corrected according to Table 2. Graph show mean ± SD. Non-parametric statistical testing was used because in each comparison at least one group is not normally distributed (Shapiro–Wilk test). Each cohort was compared to control at each milestone using a Mann–Whitney U-test; no pairs were found to be significantly different (NS). (**b**) Duration of the second embryo cycle (calculated as t3-t2), third cell division (calculated as t5-t3) and blastulation (calculated as tB-tSB). Graph shows mean ± SD. Non-parametric statistical testing was used because in each comparison at least one group is not normally distributed (Shapiro–Wilk test). Each cohort was compared to control for each event using a Mann–Whitney U-test; no pairs were found to be significantly different (NS). (**c**) Second cell division event. Linear regression analysis of the relationship between duration of cleavage (calculated as t3-t2; y axis) and synchronicity between the two cleavage events (calculated as t4-t3; x axis). A separate linear regression model was generated for each cohort, and these indicated a significant relationship within each group. Individual data points are plotted and colour-coded by cohort. Lines indicate linear regression equation. Shading indicates 95% confidence interval of model. Control, n = 352; murmur, n = 24; mild, n = 11; complex, n = 7. *p*-values indicated in legend. (**d**) Third embryo cycle. Synchronicity between the four cleavage events (calculated as t8-t5). Graph shows mean ± SD. A significant reduction is observed in the mild CHD cohort relative to controls (Mann–Whitney U-test, *p* = 0.016; *), but no difference is seen in the other cohorts (ns).

During cleavage stage embryonic development, typically, the oocyte divides to produce a 2-cell embryo during the first embryo cell cycle (ECC). The second embryo cell cycle (EEC2) results in a 4-cell embryo and EEC3 results in an 8-cell embryo, although anomalous divisions are not uncommon. We examined the endpoint of each of these cell divisions (2-cell stage, 4-cell stage and 8-cell stage), together with time of pronuclei fading, and the time to reach morula and blastula stages. No significant difference was observed between any CHD cohort and controls (Figure 3a).

Next, we looked at the duration of cleavage within each embryo cell cycle. For ECC2, this is calculated as the time from the 2-cell to 3-cell stage (t3-t2). The same figure for EEC3 is calculated by taking the time from the 3-cell to 5-cell stage (t5-t3) [21]. This is because the third cell division of the earliest blastomere begins before the second of the last is complete. No significant difference was observed between any CHD cohort and controls (Figure 3b).

The duration of blastulation is defined at the time from the start of cavitation to formation of a full blastocyst (tB-tSB) [30]. Again, no significant difference was observed between any CHD cohort and controls (Figure 3b).

Each ECC involves a number of individual cell cleavage events. For example, during EEC2, each of the two blastomeres of the 2-cell embryo undergoes cleavage, resulting in a 4-cell embryo. The timing of the two individual mitotic events during EEC2 and of the four events during EEC3 are not always synchronised, and it is possible to see the emergence at different timepoints, for example, of the 5th, 6th, 7th and 8th cells during EEC3. Thus, we can measure the synchronicity of cell cleavage events across each cycle by comparing how soon after the first cleavage each subsequent one appears. This is measured as t4-t3 for EEC2 and t8-t5 for the EEC3 [30]. Lower synchronicity values indicate more synchronised cell cleavage events.

We examined the relationship between the duration of cleavage and the synchronicity of cleavage during ECC2 (from 2 cells to 4 cells) using linear regression analysis. A separate linear regression model was made for each cohort (Figure 3c). In each case, a significant (*p* < 0.05) negative relationship was seen, indicating that embryos which take longer to reach the 3-cell stage subsequently progress more quickly to the 4-cell stage.

The regression lines generated for the complex CHD (red) and mild CHD (green) cohorts were very similar and appeared to have a steeper gradient than controls (suggesting embryos reaching the 3-cell stage at the same time as controls would reach the 4-cell stage more quickly), while the murmur cohort appeared to have a shallower gradient (Figure 3c). However, a linear regression model, including all cohorts, did not find a significant interaction (slope *p* = 0.16; intersection, *p* = 0.96), indicating no significant between cohorts.

We performed a similar analysis for the third cell division (from 4 cells to 8 cells), but we were unable to derive a linear regression model to explain the data within the three CHD cohorts (data not shown). However, pairwise comparison of third cell division synchronicity values for each cohort against controls revealed a significant increase in synchronicity (reduced time difference) for the mild CHD group only (Figure 3d). This result suggests that embryos are progressing to the 8-cell stage more quickly in this group.

Thus, while our analysis suffers from low power due to the small size of the CHD cohorts, the data from both the second and third cell division events point in the same direction and suggest that cell cleavage events may be more synchronised between the blastomeres of the embryos resulting in CHD affected live births.

### 3.5. CHD Embryo Development Against Matched Unaffected Controls

In our second analysis, we paired each embryo which resulted in a live birth affected with CHD with a single unaffected control, matching as closely as possible the method of fertilisation, clinic at which the procedure was performed, age and dates. Details of matching criteria are given in the methods.

We first examined the time to reach the developmental milestones described above. In order to allow a direct comparison between IVF- and ICSI-derived embryos, we again corrected values for IVF-derived embryos using the values shown in Table 2.

**Figure 4:**
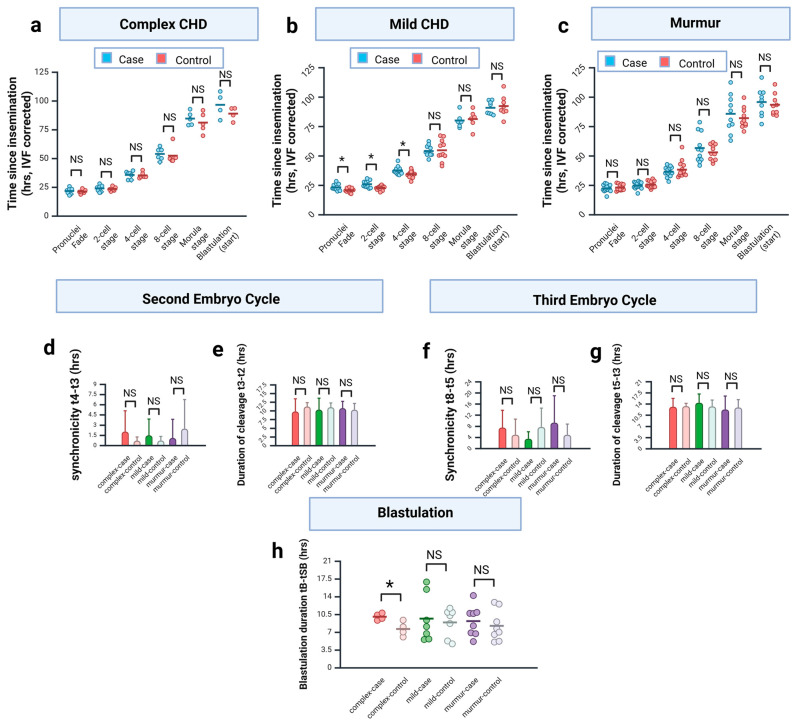
Comparison of CHD embryos to matched controls reveals specific changes. (**a**) Complex CHD group. Time from insemination to key developmental milestones. IVF-derived embryo values corrected. Only cases for which data available for paired control included in analysis. Graph shows individual data points; bar indicates mean value. Data were tested for normality (Shapiro–Wilk test) and then assessed using either a paired *t*-test (tPNF, t2, t4, tM, tSB) or Wilcoxon matched pairs signed-rank test (t8), as appropriate (two-tailed). No significant difference was seen at any milestone (NS). (**b**) Mild CHD group. Time from insemination to key developmental milestones. IVF-derived embryo values corrected. Only cases for which data available for paired control included in analysis. Graph shows individual data points; bar indicates mean value. Data was tested for normality (Shapiro–Wilk test) and then assessed using a paired two-tailed *t*-test (* *p* < 0.05; NS = not significant). (**c**) Murmur group. Time from insemination to key developmental milestones. IVF-derived embryo values corrected. Only cases for which data available for paired control included in analysis. Graph shows individual data points; bar indicates mean value. Data were tested for normality (Shapiro–Wilk test) and then assessed using either a paired *t*-test (tPNF, t2, t8, tM, tSB) or Wilcoxon matched pairs signed-rank test (t4), as appropriate (two-tailed). No significant difference was seen at any milestone (NS). (**d**–**g**) Second and third embryo cycles. Graph shows mean ± SD. Only cases for which data available for paired control included in analysis (t8-t5: complex n = 6; murmur n = 11; t5-t3: complex n = 6). Data was tested for normality (Shapiro–Wilk test) and then assessed using either a paired *t*-test (t5-t3) or Wilcoxon matched pairs signed-rank test (others), as appropriate (two-tailed). No significant difference was seen. (**h**) Blastulation. Graph shows individual data points; bar indicates mean value. Only cases for which data available for paired control included in analysis (complex n = 4; mild n = 7; murmur n = 8). Data were tested for normality (Shapiro–Wilk test) and then assessed using a paired two-tailed *t*-test (* *p* = 0.016; NS = not significant).

Analysis of the complex CHD cohort revealed no significant difference between cases and matched controls at any developmental milestone examined (Figure 4a). There appeared to be a trend towards a delay in the study group embryos relative to controls at later stages such as morula and start of blastulation based on the mean values, but due to high variance within each cohort, this was not significant (Figure 4a).

In contrast, examination of the mild CHD cohort showed no evidence of delay at later stages but rather revealed a significant delay at the three earliest developmental milestones (fading of pronuclei, 2-cell stage and 4-cell stage; Figure 4b).

We noticed a very high variation between embryo morphokinetics within the murmur cohort, particularly at later developmental stages, which was much greater than either of the other cohorts (Figure 4c). No significant differences were found between cases and controls (Figure 4c).

We next examined the timings of the second and third embryo cycles. We were unable to fit a linear regression model to show the relationship between duration of cleavage and synchronicity for either division (data not shown). Instead, we performed pairwise analysis between cases and controls separately for duration of cleavage and synchronicity. This revealed no significant effect on either the second embryo cycle (Figure 4d,e) or the third embryo cycle (Figure 4f,g).

Finally, we examined the duration of blastulation (tB-tSB) between matched cohorts. This analysis revealed that blastulation took significantly longer in the complex CHD cohort against paired matched controls (Figure 4h). No effect was seen for either the mild CHD or murmur cohorts (Figure 4h).

## 4. Discussion

### 4.1. Changes in Cleavage Stage Embryos May Be Associated with Mild CHD

Perhaps the strongest evidence we found for a link between morphokinetics and CHD risk was that observed in the mild CHD cohort. In the matched control analysis, we observe a delay in reaching the early developmental mileposts of pronuclei fading, 2-cell stage and 4-cell stage (Figure 4b). While this delay was not observed in the large control analysis (Figure 3a), we do observe a significant difference in synchronicity of cell cleavage events during the third embryo cycle (Figure 3d), the transition from four cells to eight. Thus, the balance of evidence is pointing towards a difference in the very earliest cleavage events in the mild CHD cohort. A number of retrospective analyses have suggested that fertility treatment specifically increases the risk of milder, acyanotic forms of CHD [4,9,10,11].

Precisely how any change in cell cycle synchronicity could impact upon CHD remains to be determined, but there is some evidence from lower animals such as sea urchin and *Drosophila* that it can regulate embryonic patterning and cell fate decisions (reviewed in [31]). Furthermore, recent evidence has shown that the lineages of the blastomeres of the 2-cell human embryo contribute unequally to the inner cell mass of the blastocyst, and that those cells thus incorporated into the embryo proper are more likely to arise from the first cell to divide at the second cell cycle [24]. Differences in cell fate also exist between the four blastomeres of the 2-cell and 4-cell mouse embryos [32,33] which may be related to unequal distribution of maternal mRNA [32]. Thus, cell cycle timing and synchronicity are important during human embryonic development.

### 4.2. Changes in Blastulation Associated with Cyanotic CHD

Our data revealed a potential effect on the duration of blastulation in the complex CHD group which was not seen in other cohorts (Figure 4h). However, this change was significant only in the matched control analysis not the larger unmatched control analysis, and this appears to be partly due to high variation within the control group (Figure 3b). Secondly, the observed result in the matched control analysis appears to arise because the mean value for the complex-control cohort is lower than other cohorts in the analysis (including other controls) and not because of an increase in the complex-case cohort mean (Figure 4h). Finally, due to absence of control data (Supplemental Table S3), we were able to include only four cases in this analysis (A2, A3, A4, A5). Thus, this result must be treated with caution, and a larger study would be needed to verify the result.

The complex CHD cohort we identified corresponds approximately to the more severe cyanotic form of CHD. Cyanotic disease, or blue baby syndrome, results from a mixing of oxygenated and deoxygenated blood and is generally caused by a complex phenotype, including both a lesion (a hole in the heart) and an obstruction to blood flow (such as stenosis). It may also arise from misrouting of vessels. We chose to use the term complex CHD in this analysis rather than Cyanotic CHD because we cannot say for certain that the patients were cyanotic. We observed the proportion of cyanotic cases to be 21.9% (7 of 32), and this is within the range reported for the general population [1,3].

Four of the cases within the complex CHD cohort (A2, A4, A5, A6) have phenotypes which could suggest heterotaxy, that is to say, heart defects resulting from a disruption to the left–right embryonic axis. Bilateral superior vena cava (seen in cases A2 and A4) is suggestive of incorrect remodelling of the vascular system during embryonic development. This system forms in the early embryo as a symmetrical series of paired veins but develops asymmetry as the foetus grows through selective regression and anastomosis of veins [34,35]. This results in a mature circulatory system in which blood from the left side of the head and upper body is carried to the right side by the brachiocephalic vein and subsequently drains via a single superior vena cava into the right atrium. Aberrant persistence of the left vena cava and failure to form the brachiocephalic vein results in bilateral superior vena cava [34]. This phenotype is a key feature of a heart demonstrating right isomerism [35] (two right sides; a detailed description of the mouse phenotype, which is similar to the human, is shown in [36]).

The arch of aorta normally loops to the left upon leaving the left ventricle, and the descending aorta is located on the left side of the body. An aorta aberrantly located on the right side of the body (seen in cases A4 and A5) is another diagnostic feature of right isomerism [35,36]. Interruption of the inferior vena cava (case A6) is a phenotype commonly seen in another class of heterotaxy, left isomerism (embryos with two left sides) [35]. Atrial septal defect (A2 and A4) is another common feature of heterotaxy due to malformation of the atria. However, it should be noted that all of these features may also occur in the absence of heterotaxy and in isolation cannot be taken as diagnostic.

Tetralogy of Fallot (Case A3) is a cyanotic disease affecting the outflow tract of the heart in which a ventricular septal defect is accompanied by a narrowing of the pulmonary artery and misalignment of the aorta, resulting in a right to left blood shunt. Tetralogy of Fallot is most commonly caused by a deletion on chromosome 22 region q11 [37,38], where a number of disease-causing genes are located. Although the notes for Case A3 state that a genetic test was conducted and found to be negative, we do not have details of the precise nature of the test. A number of distinct 22q11 deletions can cause disease [38,39], some of which are missed by standard tests; therefore, we cannot rule out a genetic cause in this instance.

In a detailed analysis of the risk of developing specific CHD subtypes following fertility treatment, Tararbit et al. [12] did not find a strong association with heterotaxy, finding instead a greater association with outflow tract defects. A smaller but more recent study also reported a higher incidence of outflow tract defects in this population [40]. Thus, the putative high incidence of heterotaxy in our cohort (12.5% of all CHD cases), compared to the much lower incidence of outflow tract defects (3.1%), is remarkable.

Heterotaxy arises from a disruption to the left–right embryonic axis. Left-sided identity is conferred by expression of the TGF-β family morphogen NODAL in the left-sided lateral plate mesoderm in the period immediately following gastrulation (reviewed in [35,41]). Symmetry is broken at a midline organiser called the node that produces a directional fluid flow through beating of cilia resulting in asymmetric expression of NODAL. Loss of NODAL expression results in right isomerism, whilst abnormal bilateral expression results in left isomerism. This patterning defect is translated into specific failures in cardiovascular morphogenesis later in development [35,42]. Outflow tract defects are less well understood but appear to arise from defects in specification or migration of second heart field cells during the remodelling phase of heart development or by their interaction with the cardiac neural crest [43].

Both of these events occur several days after the window of development we have examined, and it is difficult to reconcile our observations with the known disease mechanisms. However, there is limited evidence to suggest that preimplantation development could impact CHD. Lineage analysis in the zebrafish embryo has shown that cells fated to become cardiac mesoderm are present in a specific pre-cardiac region of the 512 cell blastula prior to gastrulation [44]. These cells carry positional information even at this early stage of development and are fated to form either atrial or ventricular myocardium [45]. Furthermore, the precursors of Kupffer’s vesicle can also be identified at this stage [46]. Kupffer’s vesicle is the fish analogue of the mammalian node, the structure which determines the left–right embryonic axis during gastrulation. Experimental alteration of metabolite distribution on one side as early as the 8-cell embryo of the *Xenopus* embryo can alter *NODAL* expression [47].

To our knowledge there is currently no evidence for the existence of such pre-cardiac cells in the mammalian embryo until the pre-streak stage (reviewed in [48]). Nevertheless, a recent abstract describing a currently unpublished study suggests that loss of the transcription factor *TBX5* (which causes Holt-Oram syndrome, including CHD phenotypes) results in epigenetic changes within the human blastula which impact upon mesoderm specification [49].

### 4.3. CHD Incidence and Cohort Stratification

We observed a CHD incidence rate of 0.51% across all cases within our fertility treatment population. This is lower than that previously reported in a meta-analysis of this population (1.3%) [4] and means that our population incidence rate is below the range reported for the general population (0.68–1.1%) [1,2,3,4]. It is possible that this is an underestimate of the true incidence within our study group due to under diagnosis and/or under reporting on the live outcome birth report.

The mild CHD cohort, which accounted for 34.4% of cases, corresponds approximately to acyanotic forms of CHD. Such cases generally result from a single lesion in isolation, do not result in deoxygenated blood getting into the aorta and therefore result in a less severe condition which may not require surgery. However, it should be noted that stratification of the dataset is a potential source of error in this study because the level of detail provided on the live birth outcome report varies considerably between patients. For example, we made the assumption that patients described on the form as having a “Heart Defect” without further details being provided had only a mild, acyanotic form of CHD. Furthermore, several cases in the mild CHD cohort were described as having a “hole in the heart.” This term normally refers to a ventricular septal defect (VSD). When a VSD occurs in isolation it results in a left to right blood shunt, which is a relatively mild condition and does not result in cyanosis. However, if a VSD is associated with a blockage to blood flow, as occurs in Tetralogy of Fallot as described above, it can result in a cyanotic right to left shunt. Thus, the term “hole in the heart” is somewhat unreliable as a classifier, and a more detailed diagnosis would be preferable. An isolated ventricular septal defect or hole in the heart was the most commonly observed heart defect in our population, accounting for 18.8% of all CHD cases within our population.

43.8% of our affected cases had a heart murmur listed on their outcome report with no other reported problem. Presence of a heart murmur is quite common in otherwise healthy children, and most heart murmurs are found to be innocent [50]. However, this is not always the case, and in rare cases, a heart murmur may be the only indication of a serious CHD [50,51]. Therefore, in the absence of details regarding follow-up examinations, we cannot draw firm conclusions regarding the severity of disease in these patients and therefore chose to analyse these as a separate cohort.

### 4.4. Delayed Development of IVF-Derived Preimplantation Embryos

Accurate morphokinetic data is very much dependent on the ability to accurately set time zero. Care Fertility follow published guidelines [30] in setting these. In an ICSI procedure, multiple oocytes from a single patient may be injected during one session, and t0 is set as the mid timepoint for the whole session. Therefore, it is not the precise point when sperm enters an oocyte, and there may be some variation between the first and last oocyte to be injected. Similarly, during an IVF procedure, t0 is set as the time at which sperm is introduced into the dish and is not the actual time of fertilisation. In common with other researchers [20,29], we observed a delay in embryos derived from IVF procedures reaching developmental milestones in comparison to ICSI-derived embryos. In order to permit a direct comparison of IVF- and ICSI-derived embryos within mixed cohorts, we decided to apply a correction to the IVF-derived data. This is not an ideal solution and could introduce a source of error, but we felt it was necessary given the low sample size in some of our cohorts.

## 5. Conclusions

This study has identified specific and subtle changes in preimplantation embryonic development which appear to be enriched within cohorts affected by CHD. The study is exploratory in nature and limited by a low sample size; therefore, results must be treated with caution. Much larger datasets are required to further investigate these hypotheses. Whilst Care Fertility undertake quality assured time-lapse image annotation practices, human subjectivity can limit data quality and accuracy of any findings. The introduction of trained artificial intelligence methodologies to assess time-lapse images promises to improve accuracy and analytic power [52].

Several biological, clinical and environmental factors have been demonstrated to impact human preimplantation embryo morphokinetic timings, including embryo ploidy, culture medium and incubation conditions, body mass index and gamete quality [28]. Where possible, variables were minimised within this analysis and control matching; however, it is possible that this study’s findings may be artefactual. More work will be needed to validate these findings, and given the difficulties in obtaining large samples of human embryos that result in affected births, it may be that analysis of mouse embryos with specific targeted CHD-related mutations could be the way forward. Many of the genes regulating left-right patterning, for example, are known.

## Figures and Tables

**Table 1:** Details of fertility treatment of cohorts analysed in this study.

Cohort	n	Maternal Age at Oocyte Collection (yrs; Mean ± SD)	Maternal Age at Embryo Transfer (yrs; Mean ± SD)	IVF % (n)	ICSI % (n)	Donor Oocyte % (n)	Donor Sperm % (n)
Large unaffected control	352	33.8 ± 4.5	35.3 ± 4.5	21.9 (77)	78.1 (275)	14.2 (50)	8.5 (30)
Complex CHD	7	32.9 ± 6.9	34.4 ± 9.6	57.1 (4)	42.9 (3)	28.6 (2)	28.6 (2)
Complex CHD: Matched control	7	31.7 ± 2.3	31.7 ± 2.3	57.1 (4)	42.9 (3)	0 (0)	14.3 (1)
Mild CHD	11	35.9 ± 4.9	36.3 ± 5.0	45.5 (5)	54.5 (6)	9.1 (1)	0 (0)
Mild CHD: Matched control	11	34.5 ± 3.9	34.5 ± 3.9	45.5 (5)	54.5 (6)	0 (0)	18.2 (2)
Murmur	14	33.1 ± 5.6	35.8 ± 5.5	21.4 (3)	78.6 (11)	14.3 (2)	7.1 (1)
Murmur: Matched control	14	33.8 ± 4.2	33.8 ± 4.2	21.4 (3)	78.6 (11)	0 (0)	14.3 (2)

**Table 2:** Correction factor for IVF embryos. The table shows the median time (hrs after insemination) of unaffected control embryos for each developmental milestone and the delay between ICSI and IVF embryos. The delay value was used as a correction factor applied to IVF embryos in subsequent analyses.

	tPNf	t2	t3	t4	t5	t6	t8	tM	tSB	tB
**ICSI**	21.80	24.40	35.43	36.55	48.13	50.03	54.89	81.66	92.75	102.89
**IVF**	24.10	27.04	37.85	39.23	51.65	52.70	57.10	84.52	94.76	103.24
**Delay**	2.30	2.63	2.42	2.68	3.52	2.67	2.21	2.86	2.01	0.35

**Table 3:** Details of birth anomalies reported on the Care Fertility live birth outcomes report for the CHD cases.

Cohort	Case	Cardiovascular Phenotype	Other Birth Defects	Genetic/Chromosomal Testing Results
**Complex CHD**	A1	1. Congenital heart disease (details not specified) 2. Operated on at 6 months and died from bleeding out 3. Congenital heart disease was not cause of death	Not specified	1. Euploid 2. same birth mother as case C2 but derived from oocytes from different donors
	A2	1. Pan systolic murmur 2. Large atrial septal defect with no significant septum 3. Moderate to large perimembraneous ventricular septal defect measuring 6–7 cm 4. Left arch stenosis 5. Bilateral superior vena cava with normal pulmonary venous drainage 6. No restricted right or left shunt 7. No coarctation adductus arteriosis 8. Required surgical intervention at 3–4 months of age	Not specified	Not specified
	A3	1. Tetralogy of Fallot 2. Died perinatally	Numerous other conditions including kidney issues.	Chorionic villus sample tested: 1. Euploid. 2. Negative for DiGeorge Syndrome test
	A4	1. Mitral valve atresia 2. Right-sided aortic arch 3. Bilateral superior vena cava 4. Atrial septal defect with oval fossa	Not specified	Not specified
	A5	1. Right sided aortic arch 2. Left subclavian artery arises aberrantly from the right aortic arch 3. Small patent foramen ovale 4. Tetralogy of Fallot and coarctation of aorta suspected at foetal anomaly scan but not seen at birth	Born prematurely at 22 + 3 weeks	Karyotyped by microarray analysis to exclude chromosome 22 microdeletion: detected a female profile with no copy number imbalances that could be considered clinically significant
	A6	1. Interrupted inferior vena cava	Not specified	Not specified
	A7	1. CHD diagnosed at foetal anomaly scan at 23 weeks 2. Born with serious congenital heart defect (details not specified) 3. Required open-heart surgery after birth	Born prematurely at 33 weeks	May be linked to genetic/chromosomal conditions (details not specified)
**Mild CHD**	B1	Hole in heart	Born prematurely at 34 weeks	Not specified
	B2	Heart defect (details not specified)	Not specified	Not specified
	B3	Small hole in the heart (not a point of concern)	Not specified	Not specified
	B4	Heart defect (details not specified)	Not specified	Not specified
	B5	Heart murmur and small hole in the heart	Not specified	Not specified
	B6	Heart defect (details not specified)	Not specified	Not specified
	B7	Heart murmur and small patent foramen ovale Did not require surgery	Not specified	Not specified
	B8	Hole in heart at birth, but this closed naturally	Born prematurely at 28 + 1 weeks	Not specified
	B9	Hole in heart	Not specified	Not specified
	B10	Heart defect (details not specified)	Not specified	Not specified
	B11	Hole in heart	Not specified	Not specified
**Murmur**	C1	Heart murmur	Not specified	Not specified
	C2	Heart murmur	Not specified	Not specified
	C3	Heart murmur	Not specified	Not specified
	C4	Heart murmur	Not specified	Not specified
	C5	Heart murmur: likely to be innocent	Not specified	Not specified
	C6	Heart murmur	Not specified	Not specified
	C7	Heart murmur	Not specified	Not specified
	C8	Heart murmur and ectopic heartbeat	Not specified	Not specified
	C9	Heart murmur	Not specified	Not specified
	C10	Heart murmur	Not specified	Not specified
	C11	Heart murmur	Not specified	Not specified
	C12	Heart murmur	flappy larynx Born at 37 weeks	Not specified
	C13	Heart murmur	Not specified	Not specified
	C14	Heart murmur	Not specified	Not specified

Notes. Cases A1 and C2 have the same birth mother but were derived from donor oocytes from different donors.

## Data Availability

The data underlying this article are available in the article and in its online Supplementary Materials. Further inquiries can be directed to the corresponding author.

## Supplementary Materials

The following supporting information can be downloaded at: https://www.mdpi.com/article/10.3390/jcdd12090370/s1, Table S1: Table showing morphokinetics data and fertility treatment details for the large healthy control cohort, Table S2: Table showing morphokinetics data and fertility treatment details for the congenital heart disease cohort and Table S3: Table showing morphokinetics data and fertility treatment details for the matched control cohort.

## Abbreviations

The following abbreviations are used in this manuscript:

CHD	Congenital Heart Disease
ICSI	Intracytoplasmic Sperm Injection
IVF	*In vitro* fertilisation
